# Curtailing Carbon Usage with Addition of Functionalized NiFe_2_O_4_ Quantum Dots: Toward More Practical S Cathodes for Li–S Cells

**DOI:** 10.1007/s40820-020-00484-4

**Published:** 2020-07-11

**Authors:** Ning Li, Ting Meng, Lai Ma, Han Zhang, JiaJia Yao, Maowen Xu, Chang Ming Li, Jian Jiang

**Affiliations:** 1 School of Materials and Energy, and Chongqing Key Lab for Advanced Materials and Clean Energies of Technologies, Southwest University, No. 2 Tiansheng Road, BeiBei District, Chongqing, 400715 People’s Republic of China; 2 School of Physical Science and Technology, Southwest University, No. 2 Tiansheng Road, BeiBei District, Chongqing, 400715 People’s Republic of China

**Keywords:** Carbon usage reduction, NiFe_2_O_4_ quantum dots, Additive substitute, Practical S cathode, Li–S cells

## Abstract

Smart combination of manifold carbonaceous materials with admirable functionalities (*like* full of pores/functional groups, high specific surface area) is still a mainstream/preferential way to address knotty issues of polysulfides dissolution/shuttling and poor electrical conductivity for S-based cathodes. However, extensive use of conductive carbon fillers in cell designs/technology would induce electrolytic overconsumption and thereby shelve high-energy-density promise of Li–S cells. To cut down carbon usage, we propose the incorporation of multi-functionalized NiFe_2_O_4_ quantum dots (QDs) as affordable additive substitutes. The total carbon content can be greatly curtailed from 26% (in traditional S/C cathodes) to a low/commercial mass ratio (~ 5%). Particularly, note that NiFe_2_O_4_ QDs additives own superb chemisorption interactions with soluble Li_2_S_n_ molecules and proper catalytic features facilitating polysulfide phase conversions and can also strengthen charge-transfer capability/redox kinetics of overall cathode systems. Benefiting from these intrinsic properties, such hybrid cathodes demonstrate prominent rate behaviors (decent capacity retention with ~ 526 mAh g^−1^ even at 5 A g^−1^) and stable cyclic performance in LiNO_3_-free electrolytes (only ~ 0.08% capacity decay per cycle in 500 cycles at 0.2 A g^−1^). This work may arouse tremendous research interest in seeking other alternative QDs and offer an economical/more applicable methodology to construct low-carbon-content electrodes for practical usage.

**Electronic supplementary material:**

The online version of this article (10.1007/s40820-020-00484-4) contains supplementary material, which is available to authorized users.

## Highlights

- Using NiFe_2_O_4_ quantum dots (QDs) as additive substitutes, the total carbon content in cathodes is sharply reduced from original ~ 26% (in traditional S/C cathodes) to a low mass ratio of ~ 5%.
- The as-built S@CB ⊆ QDs demonstrate more appropriate tap density (~1.32 g cm^−3^) and specific surface area (~19.9 m^2^ g^−1^) values than S@CB counterparts.
- NiFe_2_O_4_ QDs additives possess superb chemisorption interactions with Li_2_Sn molecules and proper charge-transfer/catalytic features to strengthen redox kinetics of overall cathode systems.

**Electronic supplementary material:**

The online version of this article (10.1007/s40820-020-00484-4) contains supplementary material, which is available to authorized users.

## Graphical abstract

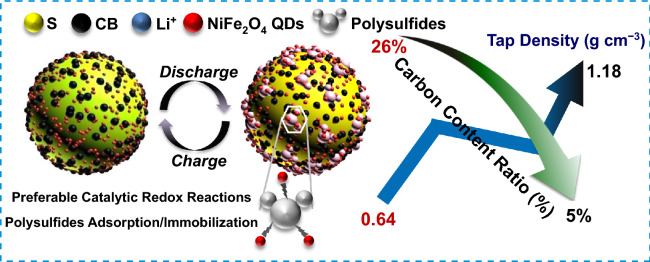

## Introduction

Increasing demands for emerging electric vehicles and surplus electricity storage have trigger searches for next-generation energy-storage systems. Nowadays, Li–S cells are extremely admirable and encouraging given their great theoretical gravimetric/volumetric capacities, good environmental benignity, and pretty low cost due to abundant natural reserves of S [1–3]. Unfortunately, their practical applications are put off by three major constraints including: (i) inferior conductivity of either S/Li_2_S cathodes or their intermediate/end-discharge products (causing sluggish redox reaction kinetics and less actives utilization ratio), (ii) huge volume expansions (e.g., ≈ 80% for S cathode) and notorious Li dendrite formation (undermining their long-term electrochemical stability/cyclic lifespan), and (iii) intractable polysulfide dissolution in organic electrolytes (inducing irreversible capacity decay and unstable Coulombic efficiency) [4, 5]. Aimed at tackling the above challenges, one prioritized strategy is the combination of functionalized carbonaceous frameworks (made up of hierarchically porous graphene, biomass-derived microporous carbon, or metallic organics [6–8]) with S or Li_2_S to offset the electrode conductivity and operation stability [9, 10]. However, once the amount of such carbon fillers reaches a critical value of 50 wt% in cathodes, there would give rise to many troublesome issues for real applications [11]. Primarily, overusing carbons with large specific surface areas (100–1500 m^2^ g^−1^) and low tap density (0.1–0.3 g cm^−3^) results in a sponge-*like* cathode that requires flooded electrolyte to sufficiently wet all electrode regions. This predicatively intensifies the electrolyte-to-sulfur (E/S) ratio and battery weight, thereby diminishing the total cell specific energies [12]. Additionally, carbons’ hydrophobicity is adverse to organic electrolyte wettability, resisting Li^+^ transport at the solid–liquid interface and deteriorating the cell kinetics. Standing on internal chemical interactions, nonpolar carbon species are incompetent to work with polar polysulfides for long cyclic stability and capacity retention [13]. For commercial concerns of Li–S cells, scientists require to introspect shortcomings in current cell technology and devote to seeking for other feasible/rational solutions.

The use of functionalized quantum dots (QDs) with a typical sub-30 nm size (comparable to that of carbon black conducting agent) may be a better option due to their great volume-to-surface ratio, far less invalid pore volumes, high specific surface energy, and enriched dangling bonds beneficial for chemical adsorption. For example, Xu et al. once employed a very small amount of black phosphorus QDs as electrocatalysts, which can adsorb soluble polysulfide intermediates and meantime promote their conversions into solid-state Li sulfides due to numerous catalytic anchors on QDs edges. The integrated hybrid cathodes showcase rapid reaction kinetics without evident shuttling effects, showing a slow capacity decay rate (~ 0.027% per cycle) among all 10^3^ cycles [2]. Park et al. reported the addition of graphene QDs into S cathodes can induce the formation of hierarchically functionalized S/carbon hybrids aided by involved O-rich groups on QDs. The robust C-S bonding can minimize irreversible losses of polysulfide anions, enabling good capacity retention (~ 1000 mAh g^−1^ after 100 cycles at 0.5 C) and fast charge-transfer behaviors (remarkable discharge capacity of ~ 540.17 mAh g^−1^ at 10 C) [14]. Hence, alternatively utilizing QDs would be highly useful for Li–S cells operation, which holds great promise in reducing carbon usage amount, thus lowering the electrolyte consumption and ensuring gravimetric/volumetric specific energies of devices.

Some transitional metal oxides have been paid special attention as both physicochemical adsorbers for Li polysulfide intermediates and good catalysts to boost long-chain Li_2_S_n_ (*n* = 3–8) conversion into insoluble species and meantime accelerate substance transitions forward solid-date Li_2_S/Li_2_S_2_ [15, 16]. Ferruginous oxide families (e.g., FeO, Fe_2_O_3_, Fe_3_O_4_), the most economical and welcome material candidates, own favorable chemical bonding to polysulfide molecules and obey reversible adsorption/desorption mechanism based on strong polar surface or *Lewis* acid—base interactions [17–19]. Another recognized prototype material should be designated to Ni-based oxides (e.g., Ni_2_O_3_, NiO, β-NiOOH), which can speed up redox reaction kinetics aided by their superb electrocatalytic activities deriving from their surface/sub-surface defects or vacancies [20–22]. As a mixed combination of Fe and Ni oxides, NiFe_2_O_4_ might be a better choice than aforementioned since it would not only anchor dissociative polysulfide species and suppress the adverse “shuttling effect” during charge/discharge procedures, but also promote cell kinetics thanks to its positive catalytic properties (e.g., expediting sulfide redox couples S^2−^/S_n_^2−^ conversion) [23]. The extra incorporation of semi-conducting NiFe_2_O_4_ (conductivity: 74.32 S cm^−1^) into cathode systems would further reinforce the electrons-transfer capability of entire electrode systems.

We herein attempt to minimize the total carbon usage by alternative use/addition of multi-functionalized NiFe_2_O_4_ QDs with a characteristic fluorescence effect at 568 nm (excitation wavelength: 325 nm, see Fig. S1) into cathode systems, aiming at building more efficient and practical Li–S cells. The implanted NiFe_2_O_4_ QDs are able to serve as “modular building blocks” in optimized electrodes for Li_2_S_n_ localization/catalysis because of their good electrical conductivity and ample anchoring sites on external surface. The as-configured cathodes would not only own a proper ability of inherent chemisorption/interactions with polysulfides but also fast charge-transfer and redox reaction kinetics. Particularly, note that by choosing NiFe_2_O_4_ QDs as additive substitutes, the overall carbon content in cathodes is reduced to a minimal level of 5%, avoiding the excessive electrolyte consumption and guaranteeing specific energy parameters. To affirm such functionalities of NiFe_2_O_4_ QDs, we have deliberately evaluated cell performances in electrolyte solutions without extra addition of LiNO_3_. As confirmed, QDs-involved cathodes showcase a great specific capacity of 921.1 mAh g^−1^ at 0.2 A g^−1^, decent rate capability (remaining 526 mAh g^−1^ at 5 A g^−1^), and very impressive cyclic stability over 500 cycles (capacity decay rate: 0.08% per cycle at 0.2 A g^−1^; almost all Coulombic efficiencies beyond 96%). The concept of “building cathodes of Li–S cells with cost-effective, durable and versatile QDs” may arouse global research interest in designing well-fitted/compatible mixed metal oxide species and construction of low-carbon-content electrode for more practical Li–S cells.

## Experimental Section

### Preparation of NiFe_2_O_4_ QDs

Typically, ~ 1.62 g ferric chloride (purity > 99.99%, *Sigma*-*Aldrich*) and ~ 1.45 g nickel chloride (purity > 99.99%, *Alfa*) are dissolved into ~ 80 mL of deionized water with an ultrasonication treatment for 30 min. The resultant solution is magnetically stirred at ambient atmospheres and dripped with concentrated ammonia (*Sigma*-*Aldrich*) until the solution pH value reaches 8. After vigorously stirring for 10 min, the obtained mixture is transferred into a Teflon-lined stainless-steel autoclave and heated at 190 °C for 10 h. When cooled down to room temperature naturally, red powder samples are fetched out by centrifugation, washed with deionized water and ethanol several times, and dried at 60 °C for later use.

### Preparation of S@CB ⊆ QDs Cathodes

S@CB ⊆ QDs hybrid samples are obtained by a heat treatment toward uniform powder mixtures containing ~ 8.5 g of sublimed S (*Sigma*-*Aldrich*), ~ 0.5 g carbon black (CB; *Ketjenblack EC*-*300* *J*), and ~ 1 g of as-made NiFe_2_O_4_ QDs at 165 °C for 12 h. For cathode fabrication, such hybrid powders are carefully grinded, mixed with PVDF (*Sigma*-*Aldrich*) at a mass ratio of 9:1, and dispersed into moderate NMP (*Fluka*, 40 mg mL^−1^) to form homogeneous slurry. The calculated S ratio for S@CB ⊆ QDs cathodes is 76.5% in theory (if excluding the polymer binder mass, the theoretical S ratio should be ~ 85%). The slurry is then pasted onto an aluminum foil and dried at 60 °C for 10 h in a vacuum oven. For comparison study, the counterpart of S@CB hybrids is also prepared using the same procedures without the addition of NiFe_2_O_4_ QDs.

### Materials Characterization and Electrochemical Testing

Fluorescent properties for QDs are measured by using a fluorescence spectrophotometer (FluoroMax-4, HORIBA, Japan). Phase structures of specimens are characterized by X-ray powder diffraction (XRD; D8 Advanced diffractometer with Cu Kα radiation, λ = 1.5418Å). The subtle geometric morphology and crystalline structure are detected by field emission scanning electron microscopy (FESEM, JEOL JSM-6700F) and transmission electron microscopy (HRTEM, JEM-2010F) equipped with energy-dispersive X-ray spectroscopy (EDS). In order to clarify the surface compositions, X-ray photoelectron spectroscopy (XPS; PerkinElmer model PHI 5600 spectrometer) is employed as well. The Raman spectra are recorded on a Renishaw 1000 NR Ar laser Raman spectroscope (532 nm laser) at ambient atmospheres. Thermogravimetric (TG; SDT600, USA) is also performed to determine the mass content under N_2_ atmospheres. Tap density values for synthesized samples (S@CB ⊆ QDs, S@CB, NiFe_2_O_4_ QDs) are determined by a tapping tester (JF-20, Xiamen, China; vibration frequency: 250 tap min^−1^; amplitude: 3 mm; total counter: 8000 times). Other tap density parameters for commercial CB (0.25 mg cm^−2^) and sublimed S powders (1.2 mg cm^−2^) are directly obtained from reagent suppliers. All 2032-type coin cells are assembled with a cathode (S@CB ⊆ QDs or S@CB), a Li foil anode, and a separator (Celgard 2300 membrane, purchased from *Sigma*-*Aldrich*) in an Ar-filled glovebox (Mikrouna Super; H_2_O < 0.1 ppm, O_2_ < 0.1 ppm). The electrolyte for Li–S cell testing is 1 M lithium bis-(trifluoromethanesulfonyl)imide dissolved in 1,3-dioxolane and dimethoxymethane (1:1 by volume) solvent. To clarify the actual S loading of tested electrodes (size: 12 mm), we have purposely measured the involved S content toward electrode specimens in the same batch. The actual S mass loading is eventually determined to be ~ 4.7 ± 0.1 mg per cell by a thermal treatment. The electrolyte/sulfur (E/S) ratio herein is measured to be a central value of ~ 4.26 μL mg^−1^. On a CS310 electrochemical workstation, the cyclic voltammetry (CV) test is conducted between ∼ 1.6 and ~ 2.8 V at a scan rate of ∼ 0.1 mV s^−1^, and the electrochemical impedance spectroscopy (EIS) measurements are carried out from 100 kHz to 0.1 Hz. Galvanostatic charge/discharge tests are conducted at varied current densities within a cutoff voltage window of 1.6–2.8 V using a professional battery tester (Land, China). To check the polysulfide adsorption ability, ~ 50 mg NiFe_2_O_4_ QDs are added into 5 mL ∼ 0.3 mol L^−1^ Li_2_S_6_ solution (pre-made by dissolving Li_2_S and S with a molar ratio of 1:5 into dimethoxymethane under vigorously stirring at 80 °C).

## Results and Discussions

### Basic Morphological and Structural Characterization

**Fig. 1:**
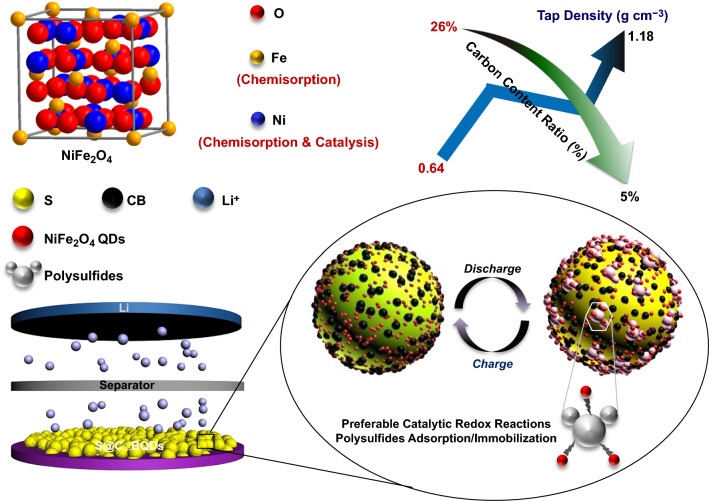
General schematics showing configured devices of (−)Li//S@CB ⊆ QDs(+) and the working mechanism of NiFe_2_O_4_ QDs

The schematic illustration in Fig. 1 manifests the general working mechanism of NiFe_2_O_4_ QDs in cathodes for Li–S cells. Generally, current mainstream materials combined with S or Li_2_S actives are still various bulky frameworks/matrix made up of hierarchically porous graphene, biomass-derived microporous carbons or high-specific-surface metallic organics. However, these S reservoir scaffolds are proven imperfect for actives loading due to (i) overmuch presence of spatial void places that are doomed to need flooded/excess electrolyte solution for complete electrode infiltration and (ii) lack of strong/polar binding interactions for anchoring dissociative molecules. In contrast, the ternary material NiFe_2_O_4_ itself has pronounced chemisorption/catalytic activities for soluble Li_2_S_n_ intermediates, and meanwhile good electrical conductivity to compensate undesired intrinsically insulating properties of S-based actives. Moreover, its QDs form enables good organic electrolyte wettability and more exposed active spots on surface for polysulfide immobilization. In a crystalline structure of NiFe_2_O_4_ QDs, both Ni and Fe atoms can provide large quantities of chemisorption sites on surface edges to confine Li_2_S_n_ molecules via electrostatic interactions, and meanwhile Ni constituents would improve Li_2_S nucleation rate through strong-binding vacancy sites and catalytically accelerate their phase conversion. In particular, the smart use of NiFe_2_O_4_ QDs substitutes entails a fact that all involved carbon content in cathodes can be largely decreased to an extremely low level of 5%, thus forming more dense/compact electrodes rather than ones full of internal pores [24]. Practically, it is surprising to notice these functional NiFe_2_O_4_ QDs can evenly integrate with S@CB to form highly dispersive and individual units; thereby, S actives together with their derivatives yielded in charge/discharge processes would be firmly restricted nearby QDs or local positions inside cathode regions. Besides, aided by efficient electrochemical catalysis of NiFe_2_O_4_ QDs, the assembled Li–S cells can exhibit very stable cyclic behaviors even without the extra addition of LiNO_3_ into the electrolyte solution, as discussed in later sections [25, 26].

**Fig. 2:**
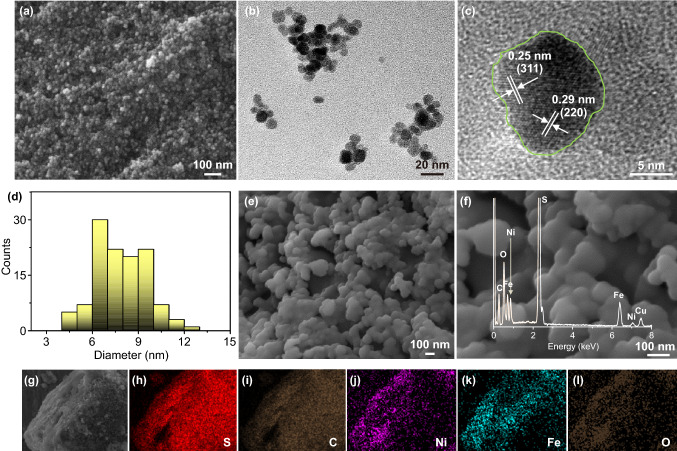
**a** Basic SEM, **b**, **c** TEM observations, and **d** size-distribution plot of NiFe_2_O_4_ QDs. **e**, **f** SEM images (inset: EDS spectrum) and **g**–**j** elemental mappings of S@CB ⊆ QDs

Then, the structural information of NiFe_2_O_4_ QDs has been examined by FESEM and TEM (Fig. 2a–c). Basic top-view FESEM and TEM observations reveal fresh NiFe_2_O_4_ QDs are evenly dispersed in the absence of any aggregations. The statistical analysis on QDs (Fig. 2d) reflects that their mean diameter size is centralized at ~ 7.88 nm within a standard deviation of ~ 1.66 nm. The well-defined crystalline lattices with spacing distances of ~ 0.25 and ~ 0.29 nm successively correspond to the characteristic (311) and (220) lattice planes of cubic NiFe_2_O_4_ (JCPDS No. 74-2081). Figure 2e, f shows typical FESEM and EDS detections on ultimate S@CB ⊆ QDs samples to uncover their delicate geometric/inherent properties. We note that such foreign NiFe_2_O_4_ QDs have perfectly coalesced with S@CB nanoparticles to form individual hybrid nano units (average size: ~ 150 nm) instead of micro bulks. The EDS spectrum (inset in Fig. 2f) and mapping results (Fig. 2g–l) further affirm the homogenous elemental distribution of Ni, Fe, O, S, and C in specimens. Their TEM observations (Fig. S2a, b) clearly reveal that there evenly distribute plenty of NiFe_2_O_4_ QDs (size: ~ 10 nm) and CB nanoparticles (size: ~ 30 nm) surrounding the S matrix, confirming the successful hybrid construction of S@CB ⊆ QDs products (though close HRTEM observations toward interfaces between NiFe_2_O_4_ QDs and S are hardly achieved due to evident S sublimation under the high-energy electron beam condition, we successfully capture the significant information on robust NiFe_2_O_4_ QDs/CB interfaces and prominent adhesive capabilities for NiFe_2_O_4_ QDs; see Fig. S2c). The XRD pattern (Fig. S3) depicts the samples’ crystallographic phase at distinct evolution stages. Obviously, diffraction peaks detected at 28.4°, 34.8°, 42.5°, 53°, and 62.3° in both NiFe_2_O_4_ QDs and S@CB ⊆ QDs samples correspond well to (220), (311), (400), (422), (511), and (440) facets of cubic NiFe_2_O_4_ (JCPDS card No. 74-2081; space group: Fd-3 m). After S infusion, intense peaks emerging at 21.1°, 22.3°, 25°, 25.9°, 27°, 27.9°, and 30.6° can be all indexed to monoclinic S (JCPDS card No. 74-2107; space group: P-21). The Raman peaks at ~ 143, ~ 202, ~ 289, ~ 548, and ~ 665 cm^−1^ (Fig. S4) successively correspond to *T*_2g_ (1), *E*_*g*_, *T*_2g_ (3) and *A*_1g_ modes for cubic NiFe_2_O_4_, keeping in line with previous literature [27–29]. Peak signals at ~ 143 and ~ 202 cm^−1^ (*T*_2g_ (1) mode) are mainly assigned to Fe–O vibrations due to the translational movement of tetrahedron atoms, while the ones at ~ 289 cm^−1^ (*E*_g_ mode) and ~ 548 cm^−1^ (*T*_2g_ (3) mode) result from symmetric bending of O with respect to Fe/Ni atoms and vibrations of octahedral groups. The symmetric stretching of Fe–O or Ni–O bonds in NiFe_2_O_4_ would also lead to a *Raman* peak at ~ 665 cm^−1^ (*A*_*1g*_ mode). The additional visible peak at a wavenumber of ∼ 464 cm^−1^ comes from the elemental S, and other two strong peaks at ∼ 1346 and ∼ 1585 cm^−1^ are ascribed to fingerprint D and G bands for CB, respectively. Besides, the TG measurement (Fig. S5) declares the total S weight percentage is up to ∼ 75 wt% in S@CB ⊆ QDs.

**Fig. 3:**
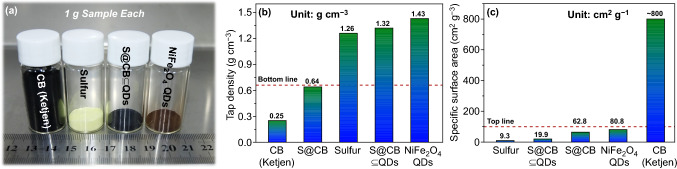
**a** A visual photograph of 1 g pre-tapped CB, S, S@CB ⊆ QDs and NiFe_2_O_4_ QDs powder samples. Parameter comparisons on the **b** tap density and **c** specific surface area of CB, S, S@CB, S@CB ⊆ QDs, and NiFe_2_O_4_ QDs

The physical density of commercial CB, sublimed S, S@CB ⊆ QDs, and pure NiFe_2_O_4_ QDs is visually compared, as presented by a photograph in Fig. 3a. All sample powders are pre-tapped for efficient materials packing. Also, their key parameters of tap density and specific surface areas are successively plotted in Fig. 3b, c. The tap density value of CB is as low as ~ 0.25 g cm^−3^, over four times less than S (~ 1.26 g cm^−3^) and NiFe_2_O_4_ QDs (~ 1.43 g cm^−3^). By contrast, the specific surface area of CB even reaches a value of ~ 800 cm^2^ g^−1^ (the standard is given by CB suppliers), far higher than that of either NiFe_2_O_4_ QDs (~ 80.8 cm^2^ g^−1^) or S (~ 9.3 cm^2^ g^−1^). Consequently, excessive use of sparse carbons is doomed to consume overmuch electrolytes to infiltrate all interior surfaces in cathodes, augmenting cells weight and lowering their specific energy. To make dense cathodes with minimized electrolyte uptake, practical S hosts are ought to own proper surface areas (< 100 m^2^ g^−1^) and tap density (0.7–1 g cm^−3^), as declared in recent literature [11]. By employing NiFe_2_O_4_ QDs as additive substitutes, the as-formed S@CB ⊆ QDs possess more appropriate tap density (~ 1.32 g cm^−3^) and specific surface area (~ 19.9 m^2^ g^−1^) values than S@CB counterparts, holing great potential in Li–S cell applications.

### Electrochemical Testing and Analysis

**Fig. 4:**
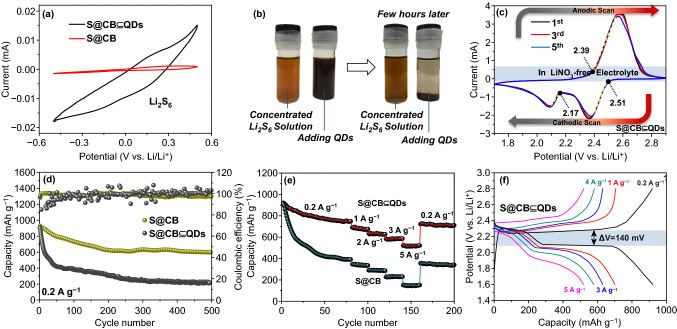
**a** CV curve comparisons between QDs-involved and QDs-free cathodes. **b** Adsorption ability of NiFe_2_O_4_ QDs in Li_2_S_6_ electrolyte. Electrochemical testing results on S@CB ⊆ QDs: **c** CV curves, **d** long-term cyclic performance, **e** rate-varied cyclic record and **f** rate-varied voltage profiles

**Fig. 5:**
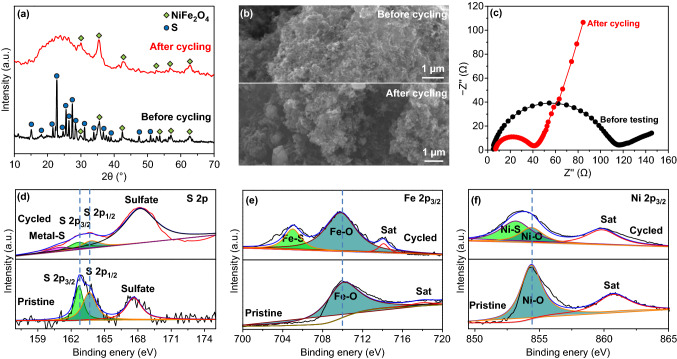
Comparison analysis of S@CB ⊆ QDs before and after 500 cycles via techniques of **a** XRD, **b** SEM, **c** EIS and **d-f** XPS: **d** S 2p, **e** Fe 2p_3/2_, and **f** Ni 2p_3/2_

The catalytic effects of NiFe_2_O_4_ QDs for polysulfide conversion kinetics are primarily probed by cyclic voltammetry (CV) method in a voltage window of − 0.5 to 0.5 V for Li_2_S_6_ symmetric cells. To exclude irrelevant influences, we also intentionally plot the CV curve of S@CB for a clear comparison study (Fig. 4a). S@CB ⊆ QDs electrodes exhibit far higher CV response than S@CB, confirming their superior electrochemically catalytic behaviors [30, 31]. This should be associated with a unique phenomenon that electropositive Fe atoms at corner sites (Fig. 1) tend to leach out by polysulfide etching, leaving rich vacant defects around Ni sites and hence facilitating/activating redox reactions [32]. An visualized adsorption experiment is further performed by adding NiFe_2_O_4_ QDs into a Li_2_S_6_ solution (∼ 0.3 mol L^−1^) to testify their absorption ability (Fig. 5b). Compared to pure concentrated Li_2_S_6_ solution, NiFe_2_O_4_ QDs-contained liquid becomes colorless after few hours, solidly certifying the excellent chemisorption capability of QDs toward Li_2_S_n_. To better confirm practical functions of NiFe_2_O_4_ QDs in full-cell operation, we have purposely excluded positive effects/contributions (e.g., on Li_2_S_n_ shuttling suppression) from additive salts and executed all cell measurements in LiNO_3_-free electrolytes. Figure 4c shows CV curves of S@CB ⊆ QDs cathodes between 1.6 and 2.8 V (*vs*. Li/Li^+^) at a scanning rate of 0.1 mV s^−1^ for initial five cycles. Cathodic peaks at ~ 2.38 and ~ 2.1 V are ascribed to the electrochemical transformation of S_8_ into long-chain polysulfide species and further reductions into solid Li_2_S_2_/Li_2_S, respectively. In subsequent anodic scans, the overlapped anodic peaks at ~ 2.58 V result from reverse complex oxidation reactions from Li_2_S_n_ (*n* = 1 − 2/4 − 8) to pristine S_8_ [33–35]. With regard to S@CB cathodes (Fig. S6), there are similar cathodic/anodic peaks but obvious potential polarization/sluggish phenomena accompanied in early cyclic periods. The onset potential positions for characteristic redox peaks of S@CB ⊆ QDs and S@CB are also labeled, respectively (see CV plots in Fig. S6). The anodic peak of S@CB ⊆ QDs is initiated at ~ 2.39 V, whereas the two-step cathodic peaks are successively lying at ~ 2.51 V (corresponding to S_8_ transformation into long-chain Li_2_S_n_ (n ≥ 4)) and ~ 2.17 V (indexed to changes from low-order Li_2_S_n_ (n < 4) to Li_2_S). Compared to S@CB cases with a higher anodic peak position of ~ 2.43 V and lower cathodic ones at ~ 2.46 and ~ 2.16 V, NiFe_2_O_4_ QDs-involved electrodes demonstrate upper electrochemical activities and lowered energy barriers for the onset of redox peaks. Such enhanced redox reaction kinetics are closely associated with intrinsic positive effects deriving from NiFe_2_O_4_ QDs, which can electrocatalytically push forward electrochemical conversions of S-based active species.

Galvanostatic charge–discharge curves of S@CB ⊆ QDs under 0.1 A g^−1^ at the 1st, 50th, 100th, 300th, and 500th cycle (Fig. S7) reveal two distinct plateaus appear at ∼ 2.38 and ∼ 2.1 V, which are in accordance to classic multi-step reductions of S_8_. The presence of a long and flat plateau at ~ 2.58 V agrees well with our former CV analysis. Even after 500 times of deep cycling, the well-defined presence of such characteristic voltage plateaus affirms the durable redox kinetics and cyclic performance of S@CB ⊆ QDs. Figure 4d shows the long cyclic records of S@CB and S@CB ⊆ QDs at 0.2 A g^−1^. S@CB ⊆ QDs hybrids can deliver a remarkable discharge capacity of ∼ 910 mAh g^−1^ and inappreciable capacity fading (only ~ 0.09% per cycle) even after 500 cycles with all Columbic efficiencies over 96%. In sharp contrast, after limited 100 cycles, S@CB cathodes exhibit only ~ 43.8% of initial capacity (∼ 908 mAh g^−1^), along with very fluctuant/unsteady Columbic efficiency records (values jumping between 82% and 109%). Given the cathode diameter is 12 mm (electrode area: ~ 1.13 cm^2^), the areal capacity value for S@CB ⊆ QDs cathodes is measured as high as ~ 3.83 mAh cm^−2^. When compared to S@CB and other metal oxide-based cathode examples (please see the added plot in Fig. S8), the areal capacity of S@CB ⊆ QDs is much higher than the value of S@CB, or even double/triple times that of counterparts (e.g., S/Co_3_O_4_/C, S/NiO/C, TiO_2_ QDs@MXene/S) [1–4].

Figure S9 shows the EIS testing results fitted by the inset *Randles* equivalent circuit. *R*_1_ in the circuit model represents the bulky resistance caused by the electrolyte phase as well as other parts of cell configurations, corresponding to the intercept with the real axis at a high-frequency range. There is no large difference in *R*_1_ between these two electrodes; the *R*_1_ value (~ 1.58 Ω) of S@CB is a bit lower than that of S@CB ⊆ QDs (~ 4.89 Ω), which may be induced by the increment of local electrolyte absorption/concentration for carbon-rich cathodes. *R*_2_ and *CPE*_1_ successively represent the charge-transfer resistance and related capacitance reflecting the kinetic nature at the solid/electrolyte interface. S@CB ⊆ QDs electrodes own a smaller *R*_2_ value (~ 114.8 Ω) than S@CB counterparts (∼ 159.7 Ω), suggesting their upper physicochemical properties aided by the addition of NiFe_2_O_4_ QDs. *W*_1_ refers to the diffusion impedance correlating with Li^+^ diffusion processes through a bulk cathode, as reflected by a tail-*like* slope at the low-frequency range [25, 36]. A larger slope of S@CB ⊆ QDs electrode indicates their superior mass-transfer behaviors for Li^+^. The corresponding Li^+^ diffusion coefficient (*D*_Li_) and double layer capacitance (*C*_d_) can be calculated according to formulas below:

1 $$D_{\text{Li}} = \frac{{R^{2} T^{2} }}{{2A^{2} n^{4} F^{4} c^{4} \sigma^{2} }}$$

2 $$C_{\text{d}} = \frac{1}{{R_{\text{ct}} }}$$

where *R* refers to the gas constant (8.3143 J K^−1^ mol^−1^), *T* is the absolute temperature, *A* is the area of electrode, *n* is the number of electrons involved in the reaction, *F* is the *Faraday* constant, *C*_Li_ is the Li^+^ concentration, *σ* is the *Warburg* factor, and *ω* is the angular velocity. Figure S10 reflects the S@CB ⊆ QDs cathode even possesses a higher *D*_Li_ (4.14 × 10^−8^ cm^−2^ s^−1^) than S@CB (0.52 × 10^−8^ cm^−2^ s^−1^), which is possibly associated with shortened Li^+^ diffusion paths or surface ion diffusivity due to less carbon usage [37]. Moreover, the *C*_*d*_ of S@CB ⊆ QDs cathode (7.3 × 10^−7^ F) is far lower than that of S@CB (1.74 × 10^−6^ F), mainly attributing to diminished specific areas of S@CB ⊆ QDs. The rate performance (Fig. 4e) is also estimated at different current rates from 0.2 to 5 A g^−1^. Apparently, the average discharge capacity of S@CB ⊆ QDs electrodes slightly drops from 910 to 691, 636, 589, and 526 mAh g^−1^ when the current rate stepwise increases from 0.2 to 1, 2, 3, and 5 A g^−1^, whose records are much better than those of S@CB (901, 352, 287, 226, and 153 mAh g^−1^). Figure 4f displays typical voltage profiles of S@CB ⊆ QDs. Specifically, S@CB ⊆ QDs cathodes exhibit relatively lower potential polarization (*ΔV *= 140 mV) when compared to S@CB cases (*ΔV *= 365 mV; Fig. S11), declaring effectively improved cell kinetics due to the addition of NiFe_2_O_4_ QDs. To further make clear effects induced by S loading ratio, basic electrochemical behaviors of cathodes with ramped S areal mass densities (including 4, 8, 10, and 12 mg cm^−2^) have been provided for comparative study (Fig. S12). Similar to the former case (~ 754 mAh g^−1^) under an areal mass of ~ 4 mg cm^−2^, S@CB ⊆ QDs cathodes (at ~ 8 mg cm^−2^) can maintain a comparable reversible capacity of ~ 702 mAh g^−1^. However, when S loading rises to a level of 10 or 12 mg cm^−2^, the evident capacity degradations of S@CB ⊆ QDs cathodes are noticed (Fig. S12a), with delivered specific capacities of ~ 465 and ~ 266 mAh g^−1^, respectively. (Corresponding specific capacity vs. mass loading plot is displayed in Fig. S12b.) This is mainly attributed to a fact that such a highly thicken/compact electrode film is not beneficial for electrolytic infiltration, leading to reduced actives utilization and deteriorated electrode operation stability (see Coulombic efficiency record in Fig. S12c; especially when S areal mass increases to 12 mg cm^−2^, huge fluctuations on Coulombic efficiency values are rather distinct).

### Postmortem Analysis on Cycled Cathodes

Postmortem analysis on cycled cathodes is systematically conducted as well. Figure 5a shows the XRD pattern of S@CB ⊆ QDs after 500 cycles. All detectable diffraction peaks accord well with those belonging to NiFe_2_O_4_ QDs, except for a broad bump-like diffraction peak at ∼ 23.3° stemming from electrolytic residuals; no other possible substances (e.g., FeS, FeO_x_, NiS, and NiO) are probed, confirming the outstanding electrochemical stability of NiFe_2_O_4_ QDs. The geometrical morphology of fatigue S@CB ⊆ QDs cathodes (Fig. 5b) clearly reveals the absence of any flower-like S crystals formation. Elemental mapping records (Fig. S13) further evidence the same uniform distribution of Ni, Fe, O element as before. The EIS testing toward S@CB ⊆ QDs cathodes before and after 500 cycles have been compared (Fig. 5c). The *R*_2_ for cycled S@CB ⊆ QDs electrodes (~ 40.3 Ω) is far lower than the value of fresh ones (~ 114.4 Ω), which might be due to remarkable reconfiguration/rearrangement of S actives in cathode regions among repeated charge/discharge processes. A higher slope suggests Li^+^ transfer rate is also enhanced due to the setup of electrochemical reaction equilibrium preferable for redox conversion. Their surface chemistry and composition are further analyzed by XPS (Fig. 5d–f). The pristine S 2p spectrum exhibits a typical spin–orbit doublet at ~ 162.7 (S 2p_3/2_) and ~ 164.1 eV (S 2p_1/2_), successively attributing to the terminal and bridging S (Fig. 5d) [38]. The additional peak situated at ~ 167.6 eV refers to sulfate species resulted from oxidation during sample synthesis. In contrast, great changes in S 2p spectrum have been observed for cycled electrodes owing to the presence of intensified peak centralized at ~ 167.3 eV, which is mostly responsible for the formation of metal (Fe or Ni)-S bonds via dangling bonds on NiFe_2_O_4_ QDs [39]. Inherent interaction protocols aforementioned are also validated by Fe 2p_3/2_ and Ni 2p_3/2_ spectra. Figure 5d compares the Fe 2p_3/2_ spectrum of S@CB ⊆ QDs cathode before and after cycling. The initial Fe 2p_3/2_ core-level spectrum (Fig. 5e) exhibits a characteristic peak (~ 710.4 eV) as well as one shake-up satellite peak (~ 719.1 eV), indicative of a typical Fe^3+^ (Fe–O bond) chemical state. After cycling, there is a novel peak arising at a lower position of ~ 705.1 eV, which is assigned to a chemical condition of Fe^2+^ (Fe-S bond). Similarly, the Ni 2p_3/2_ XPS spectrum (Fig. 5f) of cycled cathodes also shows a new peak redshifted to ~ 853.2 eV, which can be elucidated by the formation of Ni-S bond. Given the absence of crystalline phase changes on NiFe_2_O_4_ QDs judged by previous XRD analysis, we conclude that strong chemical interactions between NiFe_2_O_4_ QDs and polysulfides should be the most convincing reason to account for above results [40–43].

## Conclusions

In summary, low-cost and multi-functionalized NiFe_2_O_4_ QDs with exceptional electrolyte wettability, rich surface adsorption/catalytic sites, and good electrical conductivity have been used as additive substitutes to curtail the carbon usage and hence build more practical S cathodes for Li–S cells. The carbon content can be greatly decreased from ~ 26% (a mean percentage level in S/C hybrids) to a low value of ~ 5% for our S@CB ⊆ QDs. We also affirm NiFe_2_O_4_ QDs additives own excellent chemisorption interactions with soluble polysulfide molecules and prominent catalytic properties for Li_2_S_n_ phase conversions and meantime boost the charge-transfer capability/redox kinetics of entire cathode systems. As a consequence, the designed S@CB ⊆ QDs hybrid cathodes demonstrate outstanding rate behaviors and quite stable cyclic performance in LiNO_3_-free electrolytes (only ~ 0.08% capacity fade per cycle in 500 cycles at 0.2 A g^−1^; Coulombic efficiency stays above 96%). Our present work may have great potential for the rational design of low-carbon-content electrodes and meanwhile arise tremendous research interest in exploiting more optional/useful QDs additives for practical Li–S cell systems.

## Electronic supplementary material

Below is the link to the electronic supplementary material.

Supplementary material 1 (PDF 1414 kb)
