# Supplementary material for: Curtailing Carbon Usage with Addition of Functionalized NiFe2O4 Quantum Dots: Toward More Practical S Cathodes for Li–S Cells
Source: Nanomicro Lett. 2020 Jul 11;12:145. doi: 10.1007/s40820-020-00484-4 (PMC7770776; doi:10.1007/s40820-020-00484-4)
Supplement: Supplementary file 1 — Supplementary material 1 (PDF 1414 kb) [file 40820_2020_484_MOESM1_ESM.pdf]

Supporting Information for

Curtailing Carbon Usage with Addition of Functionalized $NiFe_2O_4$ Quantum Dots: Toward More Practical S Cathodes for Li-S Cells

Ning Li[1], Ting Meng[1], Lai Ma[1], Han Zhang[1], JiaJia Yao[2], Maowen Xu[1], Chang Ming Li[1,*], Jian Jiang[1,*]

[1]School of Materials and Energy, and Chongqing Key Lab for Advanced Materials and Clean Energies of Technologies, Southwest University, No. 2 Tiansheng Road, BeiBei District, Chongqing 400715, People's Republic of China

[2]School of Physical Science and Technology, Southwest University, No.2 Tiansheng Road, BeiBei District, Chongqing 400715, People's Republic of China

*Corresponding authors. E-mail: jjiang@swu.edu.cn (J. Jiang); ecmli@swu.edu.cn (C.M. Li)

Supplementary Figures

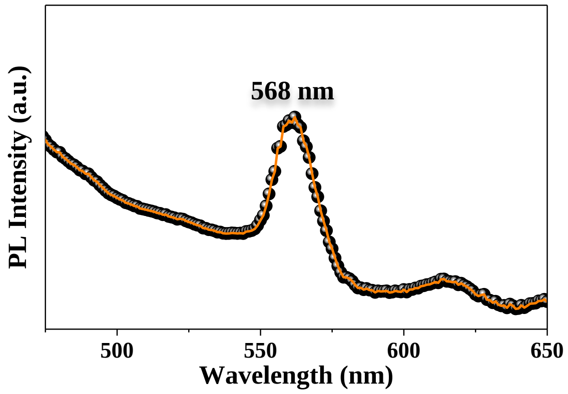

Fig. S1 Fluorescence spectrum of $NiFe_2O_4$ QDs

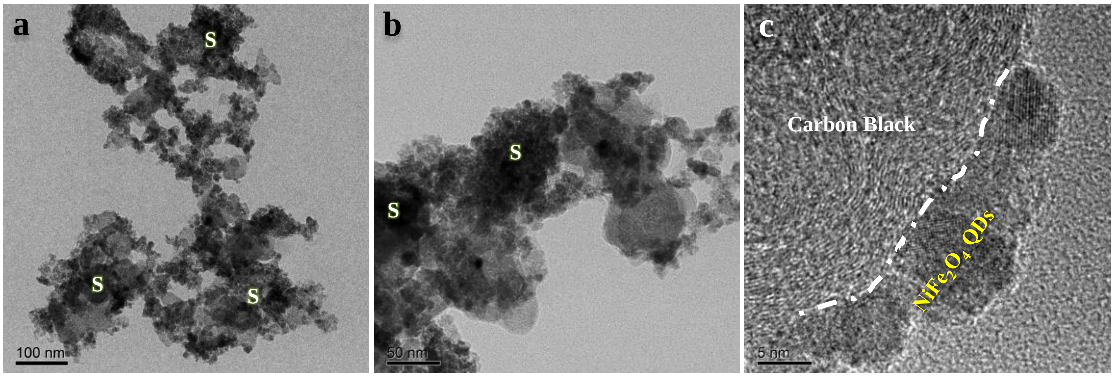

Fig. S2 Typical TEM images of S@CB⊆QDs

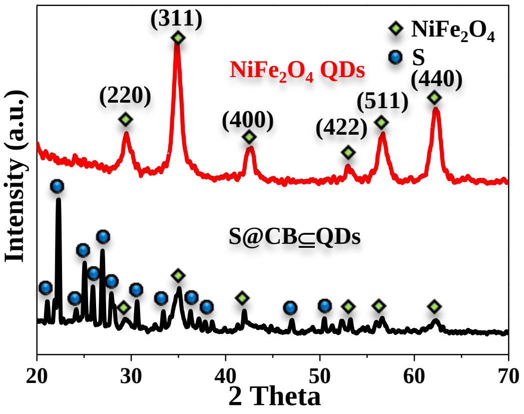

Fig. S3 XRD patterns of $NiFe_2O_4$ QDs and S@CB⊆QDs hybrids

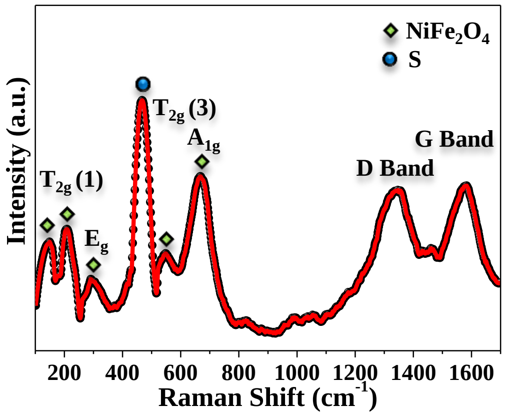

Fig. S4 *Raman* spectrum of S@CB⊆QDs

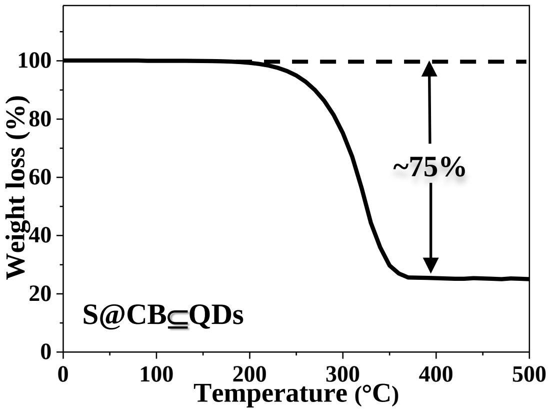

Fig. S5 TG curve of S@CB⊆QDs

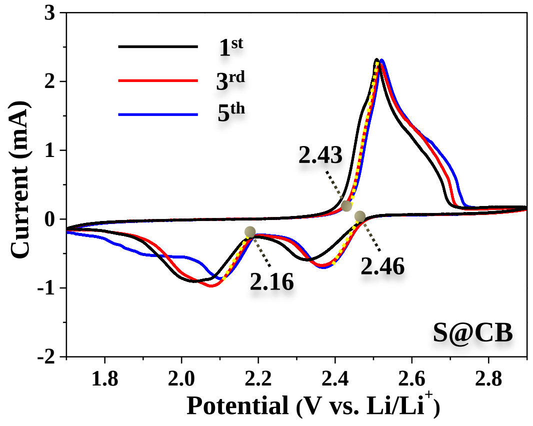

Fig. S6 CV curves of S@CB hybrid cathode

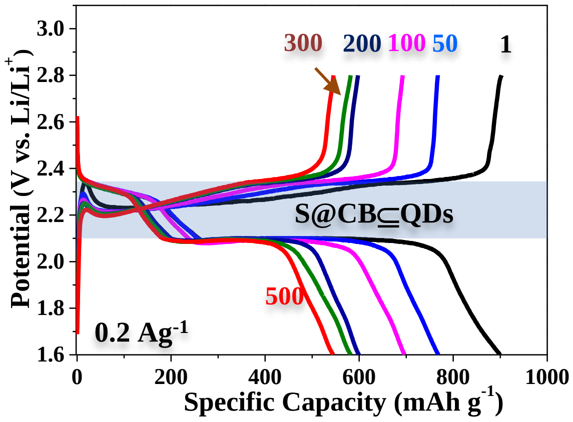

Fig. S7 Galavanostatic charge/discharge profiles of S@CB⊆QDs cathode

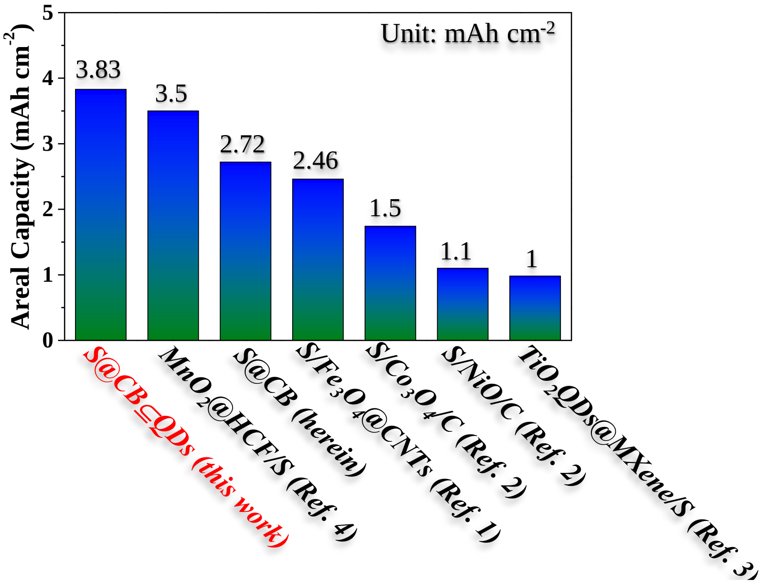

Fig. S8 Areal capacity comparison of S@CB⊆QDs, S@CB and other metal oxide-based cathodes

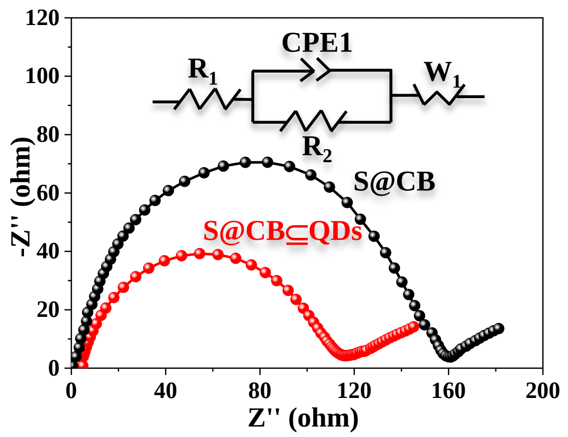

Fig. S9 The EIS plot comparison between the S@CB and S@CB⊆QDs cathodes

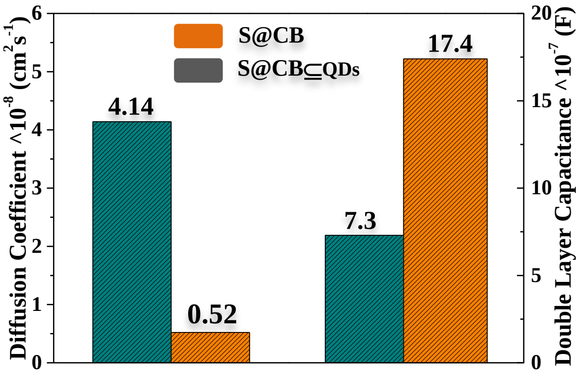

Fig. S10 Comparisons on Li^+ diffusion coefficients and double layer capacitances between the S@CB and S@CB⊆QDs cathodes

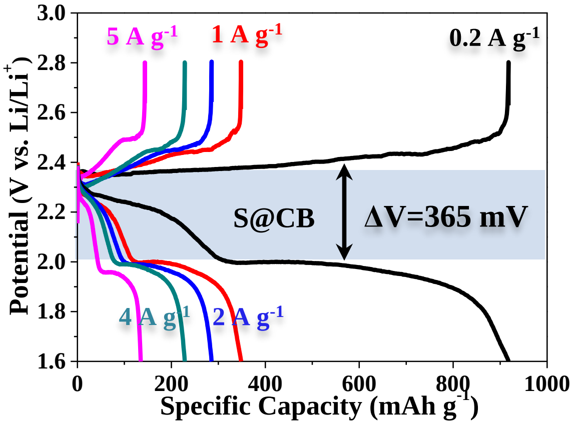

Fig. S11 Charge/discharge voltage profiles of S@CB cathodes at different current densities

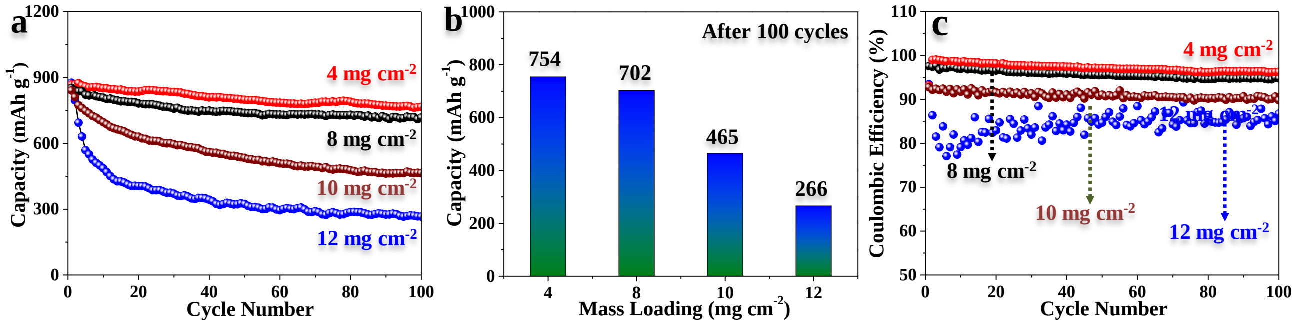

Fig. S12 a Cyclic testing of S@CB⊆QDs cathodes under different mass loadings, **b** specific capacity *vs.* areal loading plot and **c** their corresponding Coulombic efficiency

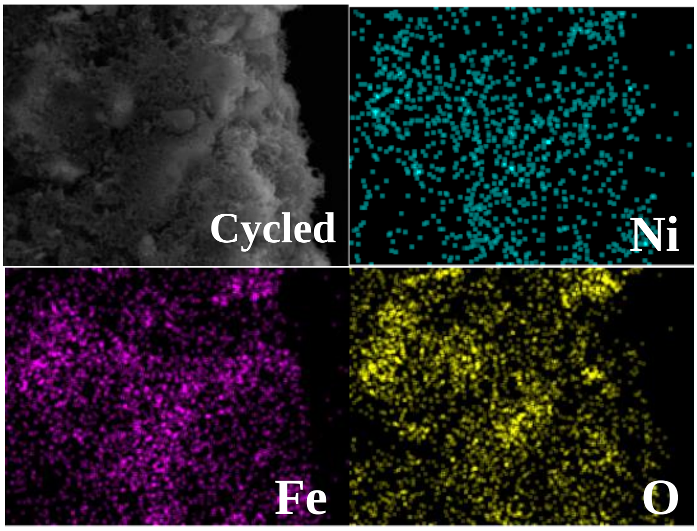

Fig. S13 EDS elemental mappings of cycled S@CB⊆QDs cathode

Supplementary References

[S1]Y. P. Zhang, R. Gu, S. Zheng, K. X. Liao, P. H. Shi et al., Long-life Li-S batteries based on enabling the immobilization and catalytic conversion of polysulfides. J. Mater. Chem. A **7**, 21747-21758 (2019). https://doi.org/10.1039/C9TA07767G

[S2]F. Ma, J. S. Liang, T. Y. Wang, X. Chen, Y. N. Fan et al., Efficient entrapment and catalytic conversion of lithium polysulfides on hollow metal oxide submicro-spheres as lithium–sulfur battery cathodes. Nanoscale **10**, 5634-5641 (2018). https://doi.org/10.1039/C7NR09216D

[S3]X. T. Gao, Y. Xie, X. D. Zhu, K. N. Sun, X. M. Xie et al., Ultrathin MXene Nanosheets Decorated with TiO_2 Quantum Dots as an Effcient Sulfur Host toward Fast and Stable Li-S Batteries. Small **14**, 1802443 (2018). https://doi.org/10.1002/smll.201802443

[S4]X. Liang, C. Hart, Q. Pang, A. Garsuch. T. Weiss, L. F. Nazar, A highly efficient polysulfide mediator for lithium-sulfur batteries. Nat. Commun. **6**, 5682 (2015). https://doi.org/10.1038/ncomms6682
